# Analytics… what’s it all about?

**DOI:** 10.1186/2052-1847-7-S1-O8

**Published:** 2015-08-11

**Authors:** Laurie Miles

**Affiliations:** 1 Head of Analytics, SAS UK and Ireland, Slough, UK

## 

This presentation aims to go over a number of things.

Firstly it will welcome the attendees to SAS UK HQ at Wittington House and give the audience a brief introduction into SAS as an organisation. This will include a short history of the company and an overview of what SAS does. This focuses on predictive analytics; the use of historical data to look for patterns and trend to essentially ‘predict the future’. This is the SAS differentiator – the use of data to not only report the past but gives insight to why things are happening, what will happen in the future and enable organisations to make fact based decisions to ensure the best outcomes.

In order to illustrate this, and demonstrate the varied uses of advanced analytics for decision making, the presentation runs then through a number of stories from both business and sport. These stories include the use of analytics to help banks reduce fraudulent transactions and make better lending decisions and well as enabling retailers to more accurately forecast sales so they can optimise their supply chains and stock levels. From the sporting world, the use of analytics to optimise performance and reduce injuries will be discussed in sports as varied as motor racing, rugby, ice hockey and rowing.

Finally, the session will give some insight and opinion as to the future of data analytics. This includes discussion around the increasing volumes of data, the increasing variety of data that needs to be analysed and the speed with which analysis needs to be made to enable real-time decision making. This section will touch upon both ‘Big Data’ and ‘The Internet of Things’.

